# Retrospective assessment of porcine circovirus 3 (PCV-3) in formalin-fixed, paraffin-embedded tissues from pigs affected by different clinical-pathological conditions

**DOI:** 10.1186/s40813-022-00293-8

**Published:** 2022-12-05

**Authors:** Àlex Cobos, Marina Sibila, Jaume Alomar, Mónica Pérez, Eva Huerta, Joaquim Segalés

**Affiliations:** 1 Unitat Mixta d’Investigació IRTA-UAB en Sanitat Animal, Centre de Recerca en Sanitat Animal (CReSA), Campus de la Universitat Autònoma de Barcelona (UAB), 08193 Bellaterra, Barcelona, Catalonia Spain; 2 Departament de Sanitat i Anatomia Animals, Facultat de Veterinària, Campus de la Universitat Autònoma de Barcelona (UAB), 08193 Bellaterra, Barcelona, Catalonia Spain; 3 OIE Collaborating Centre for the Research and Control of Emerging and Re-Emerging Swine Diseases in Europe (IRTA-CReSA), 08193 Bellaterra, Barcelona, Catalonia Spain; 4 IRTA Programa de Sanitat Animal, Centre de Recerca en Sanitat Animal (CReSA), Campus de la Universitat Autònoma de Barcelona (UAB), 08193 Bellaterra, Barcelona, Catalonia Spain

**Keywords:** Porcine circovirus 3 (PCV-3), PCV-3 systemic disease (PCV-3-SD), PCV-3 reproductive disease (PCV-3-RD), Periarteritis, In situ hybridization, Real time quantitative polymerase chain reaction (qPCR)

## Abstract

**Background:**

Porcine circovirus 3 (PCV-3) is a recently discovered pathogen of swine that has been associated with several conditions. However, many questions remain unanswered regarding its infection, especially in terms of pathogenesis and disease impact. The aim of the present study was to retrospectively investigate the presence of PCV-3 genome by real time quantitative PCR (qPCR) and in situ hybridization (ISH) on selected formalin-fixed paraffin-embedded tissues of pigs affected by different clinical conditions and histological lesions.

**Materials and methods:**

Conditions investigated included porcine dermatitis and nephropathy syndrome (PDNS), periweaning failure-to-thrive syndrome (PFTS), congenital tremors type AII, reproductive disorders, and pigs affected by systemic periarteritis/arteritis, myocarditis, or encephalitis. Studied cases (*n* = 587) were investigated from a diagnostic database (*n* = 4162) that comprised samples collected within the period 1998–2021. From each condition/lesion, 10 to 12 cases were subsequently selected and tested by qPCR and ISH (72 cases total).

**Results:**

A total of 587 cases fulfilled inclusion criteria of the different studied conditions and were distributed among the seven groups. For the further selected cases, PCV-3 genome was found by qPCR in 12/12 periarteritis, 5/10 reproductive disease, 5/10 PFTS, 3/10 myocarditis, 1/10 encephalitis and 1/10 congenital tremor cases. PCV-3 was not found in any of the PDNS cases assessed. In periarteritis cases, tissues more commonly affected were mesenteric arteries and kidney. Reproductive disease cases associated to PCV-3 genome consistently displayed myocarditis. The lesions and labelling distribution of PFTS cases with presence of PCV-3 genome were comparable to those of the periarteritis group. qPCR and ISH yielded similar results within each studied case and were statistically comparable.

**Conclusion:**

Our results suggest that periarteritis is the hallmark lesion of PCV-3-SD, and that mesenteric lymph node and kidney appeared to be the most reliable organs to confirm the presence of PCV-3 genome in cases with periarteritis.

**Supplementary Information:**

The online version contains supplementary material available at 10.1186/s40813-022-00293-8.

## Background

Porcine circovirus 3 (PCV-3) was first identified in 2015 [1] and retrospective studies have dated evidence of infection as soon as 1967 in Brazil [2], 1993 in Sweden [3] and 1996 in Spain and China [4, 5]. Ever since, this virus has been found in pigs affected by different clinical conditions and histological lesions, both under natural and experimental conditions: porcine dermatitis and nephropathy syndrome (PDNS) [1], reproductive failure [1, 6, 7], multisystemic inflammation associated to wasting piglets with or without anorexia [8, 9] and congenital tremors (CT) [10]. Interestingly, an exploratory metagenomic study of pigs revealed significant higher abundance of PCV-3 genome in piglets affected by periweaning failure-to-thrive syndrome (PFTS) compared to healthy matched ones [11]. When histopathological assessment has been available, periarteritis has been the most commonly described lesion in these reports, but PCV-3 has also been found in cases of non-suppurative encephalitis [6] and myocarditis [6, 8]. Based on existing information, two separate conditions have been proposed as being causally associated with PCV-3, namely PCV-3-systemic disease (PCV-3-SD) and PCV-3-reproductive disease (PCV-3-RD) [12].

Although the diagnostic criteria of these two PCV-3 associated diseases are well defined, their final diagnosis is still challenging for several reasons: (1) laboratory techniques able to detect the virus within lesions are restricted to in situ hybridization (ISH) [7], since no reliable antibodies to detect PCV-3 antigen in formalin-fixed, paraffin-embedded tissues do exist commercially to date. Importantly, the cost of ISH is also a major constraint for being offered as a routinary laboratory technique; (2) clinical outcomes associated to PCV-3 are rather unspecific, and many other pathogens should be considered; (3), histopathological assessment experience is a fundamental asset, since associated lesions both in PCV-3-SD and PCV-3-RD may be relatively subtle and acquaintance is needed to suspect those conditions. On the other hand, viral circulation among swine populations without evidence of disease has also been widely reported, suggesting that subclinical infection may be the most common outcome, without evidence regarding the extent of the impact on productive parameters [13, 14].

The objective of the present study was to analyse the presence of PCV-3 infection (by qPCR and ISH) in formalin-fixed paraffin-embedded (FFPE) samples from retrospective diagnostic cases of pigs showing clinical conditions and histological lesions previously associated to this virus. Additionally, the present study aimed to compare results of both genome-detecting techniques on FFPE tissues.

## Materials and methods

### Retrospective histopathological assessment of lesions compatible with PCV-3 associated diseases

Pig cases submitted to the *Servei de Diagnostic en Patologia Veterinaria* (SDPV) at the *Universitat Autònoma de Barcelona* (UAB), Barcelona, Spain, between 1998 and 2021 were investigated. The database was initially filtered and only porcine circovirus 2 (PCV-2) negative pigs were included in this study (*n* = 4,162); such negativity was determined through either ISH or immunohistochemistry (IHC), depending on the year of submission [15]. Animals were firstly classified as perinatal (aborted, stillborn and weak-born piglets less than 3-day-old piglets) and postnatal (more than 3-day-old piglets). Perinatal piglets were classified as CT or reproductive disease (RD) according to its clinical history. Postnatal piglets were classified as PDNS based on presence of characteristic histological lesions, or as periarterititis/arteritis (periarteritis from now on), myocarditis or encephalitis based on presence of such histological lesions. Within the postnatal group, a priority of lesions was established to distribute cases with more than one lesion as follows: periarteritis > myocarditis > encephalitis. All encephalitis cases were tested against Aujesky's disease virus by IHC [18], being negative. Cases previously diagnosed as PFTS were included in this study regardless of the presence of any of the histological lesions mentioned in Table 1. Since porcine reproductive and respiratory syndrome virus (PRRSV) has also been described as causing vascular lesions, an IHC study to detect this viral antigen was performed as previously described [16] for cases classified in the periarteritis and PFTS groups.

**Table 1:** Inclusion criteria and number of cases found for each clinical-pathological group

Age classification	Group	Number of cases	Inclusion criteria	Investigated target tissue/s by ISH and qPCR
Perinatal	Reproductive disease	149	History of reproductive failure (abortion, mummified, stillborn) with or without myocarditis and/or arteritis/periarteritis	Heart
	Congenital tremors	84	Newborn piglets clinically affected by this condition	Central nervous system
Postnatal	PDNS	80	Presence of characteristic systemic necrotizing vasculitis and fibrinosuppurative glomerulitis [17]	Kidney
	Arteritis/Periarteritis	40	Presence of systemic periarteritis and/or arteritis with or without other lesions in postnatal pigs (mononuclear myocarditis, non-suppurative encephalitis, interstitial pneumonia)	Any tissue displaying periarteritis
	Myocarditis	22	Presence of mononuclear myocarditis in > 3-day-old piglets and absence of systemic arteritis/periarteritis. Some cases had also other lesions (non-suppurative encephalitis, interstitial pneumonia and/or interstitial nephritis)	Heart
	Encephalitis	101	Presence of non-suppurative encephalitis in absence of myocarditis and/or systemic periarteritis. All cases were negative for Aujeszky’s disease virus detection by IHC [18]	Central nervous system
	PFTS	111	Clinical signs compatible with PFTS and presence of thymic atrophy, superficial gastritis and villous atrophy in small intestine [19]. These cases featured or not any of the lesions mentioned above	Pooled tissues

Perinatal: aborted, stillbirth or weak born < 3-day-old piglets; Postnatal: ≥ 3-day-old pigs

*PDNS* Porcine dermatitis and nephropathy syndrome, *PFTS* Periweaning failure-to-thrive syndrome

### Histopathology

All selected cases (*n* = 587, Table 1) were re-assessed histopathologically to describe the presence and severity of microscopic lesions such as non-suppurative myocarditis, interstitial pneumonia, interstitial nephritis and non-suppurative encephalitis (glial foci and perivascular cuffing). These lesions were graded as absence (−), mild (+), moderate (++) and severe (+++) accounting for the intensity of the inflammatory infiltrates and the extension of the lesions.

**Fig. 1:**
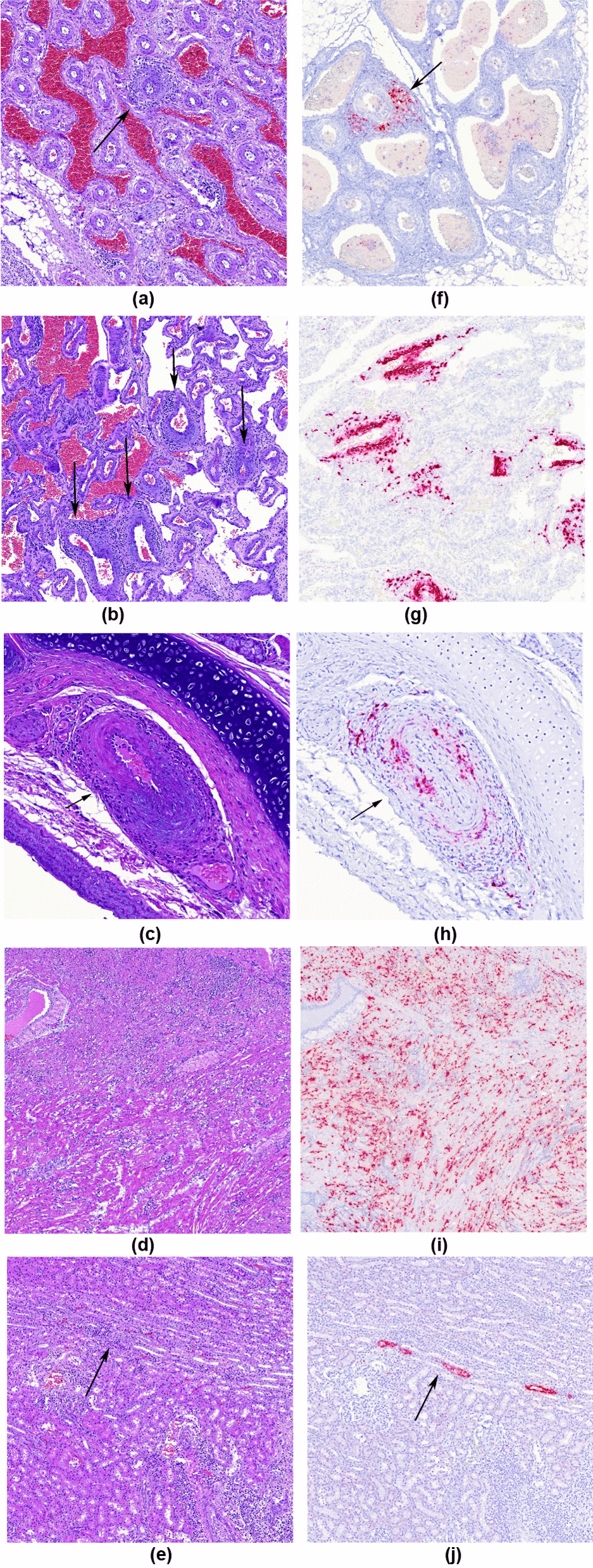
Examples of arteritis and periarteritis score, as well as other lesions: **a** Periarteritis in mesenteric arteries, H&E. Score 1 (<10% arteries affected); **b** Periarteritis in mesenteric arteries, H&E. Score 3 (>25% arteries affected); **c** Case of periarteritis within bronchial artery; note that bronchial artery (arrow) is adjacent to principal bronchi. H&E stain; **d** Moderate mononuclear lymphohistiocytic myocarditis. H&E stain; **e** Mild multifocal lymphoplasmacytic interstitial nephritis. H&E stain; **f** Periarteritis in mesenteric arteries, PCV-3 ISH. This case received a score of 1; **g** Periarteritis in mesenteric arteries, PCV-3 ISH. This case received a score of 3; **h** Bronchial artery with periarteritis where positive labelling is seen within bronchial artery muscular wall and inflammatory infiltrates, PCV-3 ISH; **i** Strong PCV-3 positive labelling (score 3) of myocardial fibres in case of moderate mononuclear myocarditis, PCV-3 ISH; **j** Positive viral genome labelling (score 1) within tubular epithelial cells of the kidney, PCV-3 ISH

Periarteritis/arteritis lesions were evaluated in a blinded fashion by two independent pathologists using a newly defined score (Table 2, Fig. 1a and 1b). Such scoring considered the number of affected small and medium sized arteries in tissues, thus it is mainly suitable for tissues with large numbers of arteries (such as kidney and mesentery). In the other studied organs, only presence (P) or absence (−) of periarterial lesions was recorded.

**Table 2:** Histopathological score for assessing arteritis/periarteritis in well-vascularized tissues such as kidney and mesentery

Score/tissue	Description
−	Absence of inflammatory infiltrates in the arterial walls or around them
+	Less than 10% of arteries affected
++	Equal or more than 10% and less than 25% of arteries affected
+++	Equal or more than 25% of arteries affected

### Subset selection for PCV-3 genome detection

A subset of 10 to 12 cases from each group (Table 1) were selected to determine the presence of PCV-3 genome in FFPE samples. These cases were selected taking into consideration the age of the animals, their farm origin, the year of submission and the lesion type and severity to maximize variability within groups (data not shown). In the RD group, cases featuring myocarditis were selected, which meant that half of them (*n* = 5) displayed this lesion. In the PFTS group, four cases were selected with periarteritis, and 6 cases without.

One to six FFPE tissue blocks were available from each case. Each one had been initially ensembled for diagnostic purposes and contained multiple tissues, pooled in similar but not always identical distribution. One FFPE block was selected for each case for PCV-3 genome detection, containing heart in myocarditis and RD cases, central nervous system (CNS) in encephalitis and CT groups, kidney in PDNS cases, and for periarteritis cases, blocks containing any tissue displaying these lesions were selected. Four FFPE tissue blocs were selected for PFTS cases, containing at least lymphoid tissues, lung, heart, kidney, liver, digestive tract (stomach and/or intestine) and CNS.

### qPCR in FFPE samples

A qPCR to quantify the amount of viral DNA was performed on FFPE material. Four tissue slices measuring 8 µm thick each were obtained and DNA was extracted as previously described [9]. In PFTS cases a single tissue slice was obtained for each one of the four selected blocs and pooled together. The qPCR was performed as described elsewhere [9]. The results obtained by qPCR were expressed in viral copies per mL of re-suspended pellet from deparaffined tissues. Samples with a viral load higher than 10^2^copies/mL of PCV-3 were considered positive and quantifiable. Samples with a viral load lower than 10^2^ copies/mL were considered positive but below quantification limit (BQL).

### *PCV3 *in situ* hibridization (ISH)*

Additional 4 µm thick cuts were obtained and then a PCV-3 ISH using RNAscope® technology was performed as described previously [7] using tissues matching the ones elected for qPCR assay. The presence of labelling was recorded semi quantitatively as described as mild ( +), moderate (++) or marked (+++), as described previously [20].

## Statistical analyses

Results from histopathological score and ISH labelling were given for each tissue assessed. qPCR results from FFPE material were expressed as viral load for the pool of tissues contained in the same paraffin block. In order to compare the results obtained with the different techniques, the highest value for ISH scoring per paraffin block was considered.

Statistical analyses were performed with cases that resulted positive for both qPCR and ISH using GraphPad Prism 9. Viral quantifications were grouped by maximum ISH score (scores 1, 2 or 3). Saphiro-Wilk test was used to assess distribution normality of qPCR results within each ISH score. The Kruskal–Wallis test with multiple comparisons (Dunn’s multiple comparisons test) was used to compare the mean PCV-3 viral load in tissues with different ISH score.

## Results

### Collection of studied cases

The arteritis/periarteritis group contained 40 cases, from which 8 corresponded to lactating piglets and 32 postweaning piglets. The main clinical concern at farm level for those cases was wasting and, to a lesser extent, anorexia, and dyspnoea. The IHC against PRRSV yielded positive results in 7 out of 40 (17.5%) cases (Additional file 1: Table S1). In these cases, positivity was restricted to macrophages in pulmonary parenchyma and spared arterial lesions in all cases.

For myocarditis, 22 cases were found, all in postweaning piglets. The concern at farm level was rather heterogeneous for this group of animals (respiratory problems, weaning, nervous signs…).

For RD, 149 cases were collected. Main concerns at farm level were abortion, mummified foetuses, and stillborn and weak born piglets.

For the rest of conditions, a total of 80 PDNS diagnosed pigs, 101 cases with en-cephalitis, 111 with PFTS and 84 with CT were histopathologically re-assessed. At farm level, the clinical signs in piglets diagnosed as PFTS and CT were compatible for these conditions. For PDNS and encephalitis the clinical history was rather heterogeny. The IHC against PRRSV in the PFTS cases always yielded negative results.

### Histopathological results

The cases selected displaying systemic arteritis/periarteritis (*n* = 40 of the periarteritis group and *n* = 7 cases with PFTS and arteritis/periarteritis), showed mainly perivascular and less frequently vascular lymphoplasmacytic to lymphohistiocitic infiltrates in media and adventitia walls of small and medium caliber arteries, found in all tissue types available except for the thymus (Fig. 1a and b). The more frequently affected vessels were the mesenteric arteries adjacent to the mesenteric lymph node (22 out of 23, 95.7%) and kidney (32 out of 41, 78.0%). Nevertheless, the lung was an exception for periarteritis, as pulmonary arteries were always spared. Periarteritis was present in a subset of lungs (6 out of 39, 15.4%) only affecting bronchial arteries, which were identified as small-sized arteries adjacent to bronchial cartilage in large-sized bronchi (Fig. 1c). Some cases additionally presented non-suppurative myocarditis (21 out of 41, 51.2%) (Fig. 1d), interstitial nephritis (8 out of 41, 19.5%) (Fig. 1e), interstitial pneumonia (16 out of 39, 41.0%) and non-suppurative encephalitis featuring gliosis (14 out of 32, 43.8%) and perivascular cuffing (9 out of 32, 28.1%). Periarteritis/arteritis in the CNS was recorded in the meningeal arteries. Within neuroparenchyma only perivascular cuffing was recorded. A summary of organs affected within the periarteritis/arteritis group can be found in Table 3. Results for each specific case can be found in Additional file 1: Table S1.

**Table 3:** Frequencies of histopathological results in different tissues for the cases within the periarteritis/arteritis group (*n* = 40)

Lesion	Organ/tissue	Total affected	Total submitted	Percentage of affected (%)
Arteritis/Periarteritis	Heart	11	35	31.4
	Spleen	16	34	47.1
	Kidney	30	35	85.7
	Liver	15	36	41.7
	CNS (meninges)	15	26	57.7
	Lymph node	16	34	47.1
	Lung	6	33	18.2
	Nasal turbinate	2	14	14.3
	Tonsil	5	31	16.1
	Stomach	7	17	41.2
	Intestine	16	35	45.7
	Mesenteric arteries	17	18	94.4
	Thymus	0	2	0.0
	Skeletal muscle	3	7	42.9
	Adrenal gland	1	4	25.0
Non-suppurative myocarditis	Heart	20	35	57.1
Interstitial nephritis	Kidney	8	35	22.9
Interstitial pneumonia	Lung	16	33	48.5
Gliosis	CNS	11	26	42.3
Perivascular cuffing		6	26	23.1

Myocarditis cases showed mild to severe lymphoplasmacytic, non-necrotizing myocarditis. Concomitant lesions recorded in this group were non-suppurative en-cephalitis (1/22, 4.5%), interstitial nephritis (7/22, 31.8%) and interstitial pneumonia (2/22, 9.1%).

RD cases showed no significative histological lesions (144/149, 96,7%) or mild to moderate non-suppurative myocarditis (5/149, 3,3%). Only one of them had characteristic multisystemic periarteritis.

PDNS cases (*n* = 80) had characteristic histological lesions featuring systemic fibrinoid necrosis of arteries’ walls and fibrino-necrotizing glomerulitis.

Encephalitis cases (*n* = 101) displayed mild-to-severe non-suppurative, non-necrotizing encephalitis characterized by multifocal to generalized glial foci and perivascular cuffing.

PFTS cases (*n* = 111) showed mild histological lesions of thymus atrophy, superficial lymphoplasmacytic gastritis and enteric villus blunting, in absence of other causes of wasting (PFTS is diagnosed by exclusion criteria [21]). Periarteritis was found in a subset of cases (7 out of 111, 6,3%), accompanied with mild non-suppurative encephalitis in 3 of these cases.

CT cases (*n* = 84) had no histological lesions or mild vacuolization of cerebellar white matter.

### qPCR and ISH results

A summary of the qPCR results and ISH scoring can be found in Table 4. Results for each case are included in Additional file 2: Table S2.

**Table 4:** Summary of qPCR and ISH results for the different studied groups

	qPCR		Maximum ISH score (number of cases)			
Group	positive cases	Mean viral load [Range]*	−	+	++	+++
Arteritis/Periarteritis	12/12	9.82 × 10^6^ [1 × 10^2^–6.05 × 10^7^]	1	1	7	3
Myocarditis	3/10	1.03 × 10^6^ [1 × 10^2^–3.08 × 10^6^]	9	0	1	0
RD	5/10	2.97 × 10^7^ [1 × 10^2^–8.10 × 10^7^]	6	0	1	3
PDNS	0/10	–	10	0	0	0
Encephalitis	1/10	6.53 × 10^2^	10	0	0	0
PFTS	5/10	2.07 × 10^6^ [1 × 10^2^—1.21 × 10^7^]	5	3	1	1
Congenital tremors	1/10	1 × 10^2^	10	0	0	0

^*^Results are given in PCV-3 copies/mL of resuspended pellet from deparaffined tissue

All cases in the arteritis/periarteritis group resulted positive by qPCR, with one of them being BQL; this latter case was negative by ISH. The labelling in the other nine was prominent in association to the arterial lesions, with scores ranging from 1 to 3, mostly in agreement with the histological score. All tissues assessed with arterial lesions had positive labelling within those damaged arteries (Fig. 1f and g); the only histological location that did not show such labelling was pulmonary arteries. Nevertheless, bronchial arteries had viral nucleic acid detected by ISH (Fig. 1h). Myocardial labelling (interstitial) matched the presence of myocarditis and was positive with high scores (2 or 3), with viral genome located both diffuse and periarterial (Fig. 1i). In lungs, PCV-3 labelling (score 1 or 2) was confined to alveolar septa and bronchial arteries, always sparing pulmonary arteries. Also, follicular centers in lymph nodes and periarteriolar lymphoid sheets (PALS) of spleen were ISH positive with variable scores (1 to 3). Kidney also had high scores (2 or 3) due to its arterial involvement, with tubular labeling being seldom observed and matching the presence of interstitial nephritis (Fig. 1j). Positive ISH results were also recorded in mesenteric arteries, almost always matching the histopathological score given. Rare labelling in the smooth muscle in the gastrointestinal tract was seen.

Three cases of the myocarditis group tested positive by qPCR, two of them being BQL. The remaining sample showed moderate ISH labelling within myocardiocytes.

For RD cases, five were positive by qPCR although one of them was BQL. Four cases (matching the ones with highest viral load by qPCR) were positive by PCV-3 ISH, with the highest scores found in the heart (labeling within myocardiocytes), spleen (viral genome located in PALS), and lymph node (follicular distribution of PCV-3 labelling). A lower ISH score (1 or 2) was observed in the lung and kidney. Labelling on arteries was scant, fitting well with the lack of periarteritis, except for one case that showed prominent periarterial labelling and evident periarteritis (the one with ISH score (3). When lung was available, pulmonary interstitial labelling was recorded as mild to moderate, and renal labelling was mild to moderate and distributed interstitially, in contrast to what was recorded in the periarteritis group.

PDNS group cases were all negative for both ISH and qPCR.

The encephalitis group revealed no labelling by ISH, also in the only positive case by qPCR (with very low viral load).

Within the PFTS group, four cases with and six cases without periarteritis were tested. All cases with periarteritis tested positive by both qPCR and ISH, but only one out of the six cases without vascular lesions was positive also by both techniques, with follicular ISH labelling mainly in the lymph nodes (score 1). The 4 cases with periarteritis displayed labelling within such lesions similarly to the cases in periarteritis group. PCV-3 labelling in CNS was focal and very mild and only present in two cases.

Congenital tremors group yielded no positivity by ISH, and only one case was positive by qPCR but BQL.

### qPCR and ISH result comparison

**Fig. 2:**
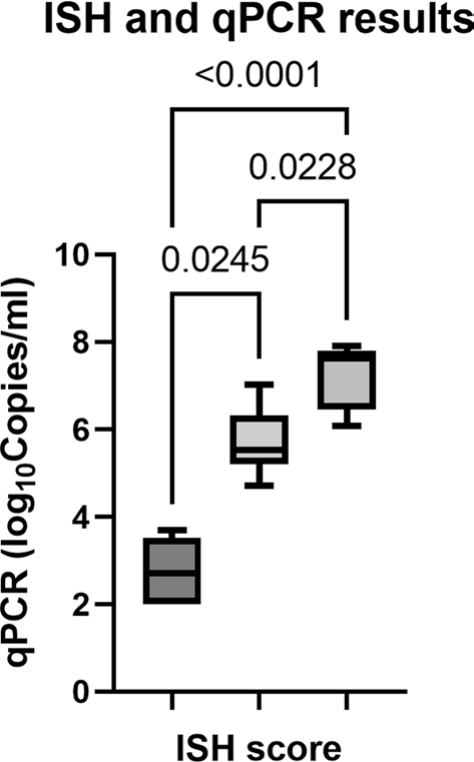
Boxplot of PCV-3 load detected by qPCR versus ISH score

Kruskal–Wallis and Dunn’s multiple comparisons tests revealed statistically significant differences between the qPCR results of cases with different ISH labelling score (Fig. 2).

## Discussion

The present study contributes to the current knowledge of PCV-3 infection, and expands existing data related with lesions apparently caused by this virus. As has been already described, arteritis and periarteritis are prominent lesions associated to PCV-3 infection in postweaning pigs affected by wasting [9, 20]. In this retrospective study, all selected periarteritis cases were positive by qPCR and all but one also for ISH. This latter case was surprising since the lesions were equivalent to those observed in the rest of submissions with periarteritis; however, the case was from 1998 and it can be speculated that time-related degradation of genetic material in paraffin embedded material may have happened; alternatively, since this was 6-month-old pig (the oldest in the series of periarteritis cases), clearance of viral particles by immune system due to already chronic lesions may have occurred. However, the obtained results strongly suggest that finding periarteritis in PCV-2-negative diagnostic cases is rather suggestive of PCV-3 infection.

A significant number of the cases with arteritis/periarteritis also featured other lesions in certain organs, such as mild to severe non-suppurative myocarditis and mild to moderate interstitial nephritis, non-suppurative encephalitis, and interstitial pneumonia. Nevertheless, the low qPCR and ISH detection of PCV-3 in myocarditis and encephalitis groups may indicate that encephalitis and myocarditis without presence of periarteritis in wasting piglets is not as characteristic as periarteritis for PCV-3-SD. The interstitial pneumonia seen in cases of PCV-3-SD was of mild severity and accompanied by low to moderate amounts of genome through ISH, which matches previous descriptions of PCV-3-SD. Its presence in cases in which PCV-2 and PRRSV infections were also excluded further supports the potential of PCV-3 in inducing interstitial pneumonia.

In the case of RD, only four cases had significant viral loads and ISH positivity, and three of them had myocarditis. On the contrary, four out of the five cases without myocarditis tested negative to both techniques. Although not fully conclusive, it appears that cases of reproductive failure with myocarditis are more prone to be associated with the presence of PCV-3 than those without myocarditis. Periarteritis was only present in one case. Noteworthy, other causes of foetal myocarditis cannot be ruled out since they were not specifically investigated.

Findings in the PFTS group suggest again an association with PCV-3 infection when periarteritis lesions are present. Presence of PCV-3 infection in absence of periarteritis was noted in only one piglet, which showed exclusively lymph node follicular labelling, which may indicate a potential subclinical infection. In cases of PFTS, all tissues were tested systematically by PCR (by pooling) and ISH. Moreover, the PFTS compatible lesions (thymic atrophy, superficial gastritis and intestinal villus blunting [19]) showed no positivity for PCV-3 by ISH. This fact suggests that PCV-3 seems not to be the direct cause of the lesions commonly described in PFTS; however, these described lesions are rather unspecific and could be derived from anorexia and wasting. Since half of the tested PFTS affected animals were positive for PCV-3, it cannot be ruled out whether this virus may play a role in the pathogenesis of this condition, as has been suggested [11].

None of the cases from the congenital tremors group resulted positive for PCV-3, except one having very low viral load measured by qPCR. Therefore, obtained results do not point out PCV-3 as a potential cause of congenital tremors. Since most of the studied cases corresponded to congenital tremors type AII, presence of atypical porcine pestivirus should be investigated as the most probable cause [22].

Overall obtained results are consistent with the proposed diagnostic criteria for PCV-3 associated diseases [12] and get insights on the potential best samples to use for diagnostic purposes. Mesenteric arteries and, to a lesser extent, kidney, are the most commonly affected tissues with periarteritis, which definitively seems to be the best “marker” of PCV-3 infection associated with lesions. Besides, other organs and tissues rich in small and medium-sized vessels (such as heart, meninges, spleen, lymph nodes, gastro-intestinal tract, and liver) may be of interest to be examined since 30 to 50% of them may also be positive in those cases displaying systemic arteritis/periarteritis. Heart was an interesting organ to be evaluated because although periarteritis in this organ are rare, interstitial myocarditis was observed in approximately half of studied cases. Existence of periarteritis in lung is rarely observed (due to the low number of bronchial arteries) but its submission to a diagnostic pathology service can be interesting to rule out interstitial pneumonia related to PCV-3, which in studied cases has been always mild.

Both qPCR and ISH yielded similar results, with ISH being unable to detect positivity on some cases with very low viral loads. Nonetheless, presence of lesion and viral genome detection in the FFPE material do not necessarily reflect overall infection severity (i.e., presence of focal periarteritis in the intestine in a paraffin block containing only an intestine section), yielding low qPCR viral load but consistent ISH labelling within the lesions. In these cases, qPCR results may not necessarily correlate with disease severity or other organs affection. To solve this issue, appropriate tissues (mesenteric lymph node and/or kidney) should be assessed for qPCR. Importantly, the availability of serum or fresh tissues may help correlating the amount of virus detected by qPCR with the presence of histological lesions compatible with PCV-3-SD and PCV-3-RD, especially when no ISH expertise is available.

One of the limitations of this study is that viral quantifications were performed in a pool of FFPE tissues, which contained variable quantities of different combinations of tissues. The lack of fresh tissue individual samples or serum from studied animals prevented the possibility to establish a potential viral load threshold for discriminating between PCV-3 subclinical infection and associated diseases. Future studies quantifying the PCV-3 load individual tissue types would be more specific and comparable to the ISH results.

Although the state of knowledge regarding PCV-3 is progressing rapidly, it is still far from reaching the existing understanding of the most well-known porcine circovirus, PCV-2 [23]. Based on a very limited number of experimental inoculations and natural infection data, proposed mechanisms of PCV-3 pathogenesis are (1) virus-induced injury, (2) type 3 hypersensitivity and (3) type 4 hypersensitivity [24], although it is yet to be determined to what extent each of these might play a role. Previous natural infection reports have shown viral genome presence in different tissues and, therefore, suggested viral replication sites in the parenchyma and in arteries of heart, lung, kidney, liver, spleen and lymph node [20], which would be in line with results of the present study. However, the specific cell types that unequivocally support PCV-3 replication are still to be defined.

Lastly, this study strongly provides retrospective histopathological and laboratory evidence of PCV-3 infection on field cases of naturally occurring disease, further supporting the recently proposed diagnostic criteria of PCV-3-SD and PCV-3-RD [12]. Cases fulfilling the PCV-3-SD and PCV-3-RD case definitions in this retrospective study dated as early as 2002, confirming that PCV-3 has not only been circulating for decades but also has been associated with reproductive disease and wasting piglets, reinforcing its putative importance on swine industry. However, in most of these cases the limited clinical history could not give clearer insights on the specific clinical profile of PCV-3 associated diseases.

## Conclusion

This study gathered retrospective data of PCV-3-SD and PCV-3-RD in diagnostic cases. Periarteritis seemed to be consistently related to PCV-3-SD, with highest amount of lesion and ISH positivity located within mesenteric arteries and kidney, which makes them excellent options for submitting for diagnosis. In this retrospective series, presence of PCV-3-SD without periarteritis was only found in one case with myocarditis and encephalitis, suggesting that periarteritis is a hallmark lesion of PCV-3-SD. Participation of PCV3 in PFTS cases was also assessed, resulting in some positive cases with characteristic features of PCV-3-SD, which could imply that some cases diagnosed as PFTS could be reclassified and diagnosed as PCV-3-SD. Regarding PCV-3-RD, our cases were mainly characterized by mononuclear myocarditis with only one of them presenting multisystemic periarteritis.

## Supplementary Information

**Additional file 1: Table S1. **Histopathological results for cases displaying arteritis/periarteritis (*n*=40) and cases of PFTS featuring periarteritis/arteritis (*n*=7), in total 47 cases.

**Additional file 2: Table S2. **Tissue availability per each studied case, including histological results and PCV-3 genome detection results (qPCR and ISH results).

## Data Availability

All data used in this review work comes from diagnostic cases pertinently archived (available but not public data) and some data can be consulted on the Additional files data attached.
