# Supplementary material for: Retrospective assessment of porcine circovirus 3 (PCV-3) in formalin-fixed, paraffin-embedded tissues from pigs affected by different clinical-pathological conditions
Source: Porcine Health Manag. 2022 Dec 5;8:51. doi: 10.1186/s40813-022-00293-8 (PMC9720923; doi:10.1186/s40813-022-00293-8)
Supplement: Supplementary file 1 — Additional file 1: Table S1. Histopathological results for cases displaying arteritis/periarteritis (n=40) and cases of PFTS featuring periarteritis/arteritis (n=7), in total 47 cases. [file 40813_2022_293_MOESM1_ESM.docx]

| Group | Case ID | Lesion | | | | | | | | | | | | | | | | | | | | |  |
| --- | --- | --- | --- | --- | --- | --- | --- | --- | --- | --- | --- | --- | --- | --- | --- | --- | --- | --- | --- | --- | --- | --- | --- |
|  |  | Periarteritis | | | | | | | | |  |  |  |  |  |  | Non-suppurative myocarditis | Interstitial nephritis | Interstitial pneumonia | Non-suppurative encephalitis | | | PRRSV status |
| Arteritis/Periarteritis |  | He | Sp | Ki* | Li | CNS | Ly | Lu | NT | To | St | In | Me* | Th | Mu | Ad |  |  |  | Perivascular cuffing | | Gliosis |  |
|  | N-861/98 | P | N | +++ | P | P | - | NS | NS | NS | - | P | +++ | NS | - | NS | ++ | + | NS | ++ | ++ | | - |
|  | N-495/02 | - | P | +++ | P | NS | - | P | NS | - | NS | - | ++ | NS | NS | NS | + | - | + | NS | NS | | - |
|  | N-660/02 | P | P | +++ | P | NS | P | P | NS | P | NS | P | NS | NS | NS | NS | + | - | - | NS | NS | | - |
|  | B-2968/02 | - | P | - | P | NS | NS | NS | NS | NS | NS | NS | NS | NS | NS | NS | ++ | - | NS | NS | NS | | - |
|  | B-802/03 | - | - | +++ | - | NS | NS | NS | NS | NS | NS | P | NS | NS | NS | NS | + | ++ | NS | NS | NS | | - |
|  | N-098/06 | P | P | NS | P | NS | P | - | NS | P | NS | P | +++ | - | P | NS | ++ | NS | - | NS | NS | | - |
|  | N-462/09 | - | NS | ++ | - | - | P | - | NS | - | - | P | NS | NS | NS | NS | - | + | + | - | - | | + |
|  | N-521/09 | - | - | + | - | NS | P | - | NS | - | NS | - | NS | NS | NS | NS | + | + | - | NS | NS | | - |
|  | N-538/10 | - | P | ++ | P | NS | P | - | NS | P | NS | P | NS | NS | NS | NS | - | - | + | NS | NS | | - |
|  | N-556/10 | - | - | +++ | - | P | P | - | NS | - | NS | P | NS | NS | NS | NS | + | - | + | - | - | | + |
|  | N-203/11 | - | P | + | - | - | P | - | - | - | NS | - | ++ | NS | NS | NS | ++ | - | - | - | - | | - |
|  | N-246/11 | P | - | + | - | P | - | - | - | - | NS | - | - | NS | P | NS | +++ | - | + | + | + | | + |
|  | N-248/11 | - | P | + | - | P | P | - | - | - | NS | - | NS | NS | - | NS | + | + | + | - | - | | + |
|  | N-273/11 | P | - | + | P | NS | P | - | - | - | P | P | NS | NS | NS | NS | - | - | - | NS | NS | | - |
|  | N-299/11 | - | - | + | - | P | - | - | - | - | - | - | NS | NS | - | NS | - | - | - | - | - | | - |
|  | B-213/15 | - | - | - | - | NS | - | - | - | NS | NS | - | + | NS | NS | NS | - | - | - | NS | NS | | - |
|  | N-131/16 | - | P | ++ | - | - | - | - | NS | NS | NS | NS | NS | NS | NS | NS | - | - | ++ | - | - | | - |

**SUPPLEMENTARY TABLE 1.** Histopathological results for cases displaying arteritis/periarteritis (n=40) and cases of PFTS featuring periarteritis/arteritis (n=7), in total 47 cases.

| Group | Case ID | Lesion | | | | | | | | | | | | | | | | | | | | |  |
| --- | --- | --- | --- | --- | --- | --- | --- | --- | --- | --- | --- | --- | --- | --- | --- | --- | --- | --- | --- | --- | --- | --- | --- |
|  |  | Periarteritis | | | | | | | | |  |  |  |  |  |  | Non-suppurative myocarditis | Interstitial nephritis | Interstitial pneumonia | Non-suppurative encephalitis | | | PRRSV status |
| Arteritis/Periarteritis |  | He | Sp | Ki* | Li | CNS | Ly | Lu | NT | To | St | In | Me* | Th | Mu | Ad |  |  |  | Perivascular cuffing | | Gliosis |  |
|  | N-409/16 | P | P | +++ | - | - | P | - | NS | P | NS | NS | NS | NS | NS | P | ++ | ++ | ++ | + | + | | + |
|  | N-105/17 | - | - | ++ | - | P | - | - | NS | - | - | - | ++ | NS | NS | - | + | - | - | + | - | | - |
|  | N-256/17 | - | P | ++ | - | P | - | - | NS | P | P | P | +++ | NS | NS | NS | - | - | + | - | - | | + |
|  | N-409/17 | - | P | + | - | - | - | - | - | - | - | - | ++ | NS | - | - | - | - | + | - | - | | - |
|  | N-440/17 | - | - | - | - | - | - | - | - | - | - | - | + | NS | - | - | + | - | - | + | - | | - |
|  | N-515/17 | - | - | + | - | - | - | - | NS | - | P | NS | + | NS | NS | NS | - | - | + | + | - | | - |
|  | N-535/17 | P | - | ++ | P | P | P | P | P | - | - | - | NS | NS | NS | NS | + | + | - | - | - | | - |
|  | N-536/17 | - | - | + | - | P | - | - | - | - | NS | - | NS | NS | NS | NS | - | - | - | - | - | | - |
|  | N-537/17 | - | - | + | - | - | - | - | NS | - | - | - | + | NS | NS | NS | + | - | + | - | - | | - |
|  | N-037/18 | - | - | - | - | P | - | - | NS | - | P | - | ++ | NS | NS | NS | - | - | - | - | - | | - |
|  | N-038/18 | - | P | + | - | P | - | - | NS | - | - | - | + | NS | NS | NS | - | - | - | - | - | | - |
|  | N-235/18 | P | - | + | P | P | P | - | NS | - | - | - | +++ | - | NS | NS | - | - | - | + | + | | - |
|  | N-310/18 | - | P | ++ | P | P | - | - | - | - | P | - | ++ | NS | NS | NS | + | - | - | - | - | | - |
|  | N-445/18 | P | - | - | P | - | - | - | - | - | P | - | NS | NS | NS | NS | - | - | + | - | - | | + |
|  | CR-215/20 5 | NS | NS | NS | NS | NS | NS | NS | NS | NS | NS | P | NS | NS | NS | NS | NS | NS | NS | NS | NS | | - |
|  | CR-215/20 14 | NS | NS | NS | NS | NS | NS | NS | NS | NS | NS | P | NS | NS | NS | NS | NS | NS | NS | NS | NS | | - |
|  | CR-215/20 33 | NS | NS | NS | NS | NS | NS | NS | NS | NS | NS | P | NS | NS | NS | NS | NS | NS | NS | NS | NS | | - |

He: heart; Sp: spleen; Ki: kidney; Li: liver; CNS: central nervous system; Ly: lymph node; Lu: lung; NT: nasal turbinate; To: tonsil; ST: stomach; In: intestine; Me: mesenteric arteries; Th: thymus; Mu: skeletal muscle; Ad: adrenal gland. P: presence of periarteritis. NS: not submitted. For non-periarteritis lesions and PRRSV immunohistochemistry, “+” means presence and “–“ means absence.

| Group | Case ID | Lesion | | | | | | | | | | | | | | | | | | | | |  |
| --- | --- | --- | --- | --- | --- | --- | --- | --- | --- | --- | --- | --- | --- | --- | --- | --- | --- | --- | --- | --- | --- | --- | --- |
|  |  | Periarteritis | | | | | | | | |  |  |  |  |  |  | Non-suppurative myocarditis | Interstitial nephritis | Interstitial pneumonia | Non-suppurative encephalitis | | | PRRSV status |
|  |  | He | Sp | Ki | Li | CNS | Ly | Lu | NT | To | St | In | Me | Th | Mu | Ad |  |  |  | Perivascular cuffing | | Gliosis |  |
| Arteritis/Periarteritis | N-193/20 | P | - | +++ | - | - | P | - | P | - | NS | P | +++ | NS | NS | NS | + | - | + | + | - | | - |
|  | N-194/20 | - | P | ++ | P | - | P | P | - | - | NS | P | +++ | NS | NS | NS | + | - | - | + | + | | - |
|  | N-249/20 | P | P | +++ | P | P | - | - | NS | - | - | P | NS | NS | NS | NS | + | + | + | + | + | | - |
|  | N-250/20 | - | P | ++ | P | P | P | P | NS | - | P | - | NS | NS | P | NS | - | - | + | + | - | | - |
|  | B-121/21 | NS | - | ++ | P | NS | - | P | NS | - | NS | NS | NS | NS | NS | NS | NS | - | - | NS | NS | | - |
|  | B-200/21 | NS | NS | NS | NS | NS | P | NS | NS | NS | NS | NS | NS | NS | NS | NS | NS | NS | NS | NS | NS | | - |
| PFTS | N-602/11 | - | - | - | - | - | - | - | NS | - | - | - | + | - | NS | NS | - | - | - | - | - | | - |
|  | N-169/12 | - | - | - | - | - | - | - | NS | - | - | - | + | - | NS | NS | - | - | - | - | + | | - |
|  | N-170/12 | - | - | - | - | - | - | - | NS | - | - | - | + | - | NS | NS | - | - | - | - | + | | - |
|  | B-230/14 | NS | NS | NS | NS | NS | P | NS | NS | NS | NS | P | NS | NS | NS | NS | NS | NS | NS | NS | NS | | - |
|  | N-224/16 | P | - | + | P | P | - | - | NS | - | - | - | ++ | NS | - | NS | + | - | - | + | - | | - |
|  | N-133/17 | - | - | - | - | - | - | - | - | - | - | - | + | NS | NS | NS | - | - | - | + | - | | - |
|  | N-134/17 | P | P | ++ | - | P | P | - | - | - | - | - | NS | NS | P | - | - | - | - | + | + | | - |
| Total affected | | 13 | 17 | 32 | 16 | 17 | 18 | 6 | 2 | 5 | 7 | 16 | 22 | 0 | 4 | 1 | 21 | 8 | 16 | 14 | 9 | | 7 |
| Total submitted | | 41 | 40 | 41 | 42 | 32 | 41 | 39 | 16 | 37 | 23 | 41 | 23 | 5 | 9 | 5 | 41 | 41 | 39 | 32 | 32 | | 40 |
| Percentage of affected (%) | | 31.7 | 42.5 | 78.0 | 38.1 | 53.1 | 43.9 | 15.4 | 12.5 | 13.5 | 30.4 | 39.0 | 95.7 | 0 | 44.4 | 20 | 51.2 | 19.5 | 41.0 | 43.8 | 28.1 | |  |
