# Supplementary material for: Retrospective assessment of porcine circovirus 3 (PCV-3) in formalin-fixed, paraffin-embedded tissues from pigs affected by different clinical-pathological conditions
Source: Porcine Health Manag. 2022 Dec 5;8:51. doi: 10.1186/s40813-022-00293-8 (PMC9720923; doi:10.1186/s40813-022-00293-8)
Supplement: Supplementary file 2 — Additional file 2: Table S2. Tissue availability per each studied case, including histological results and PCV-3 genome detection results (qPCR and ISH results). [file 40813_2022_293_MOESM2_ESM.docx]

**SUPPLEMENTARY TABLE S2.** Tissue availability per each studied case, including histological results and PCV-3 genome detection results (qPCR and ISH results).

| Group | Case ID | Year submitted | Age | Tissue | Periarteritis | Other lesions* | ISH score | qPCR viral load** |  |
| --- | --- | --- | --- | --- | --- | --- | --- | --- | --- |
| Periarteritis | N-861 | 1998 | 6m | He | P | + | - | 1.00X10^2^ |  |
|  | N-660 | 2002 | 2m | Ki | +++ | - | ++ | 1.84X10^6^ |  |
|  |  |  |  | He, In, Sp | P | - | ++ |  |  |
|  | N-98 | 2006 | 20d | Sp | P | - | +++ | 6.05X10^7^ |  |
|  |  |  |  | He | - | ++ | +++ |  |  |
|  |  |  |  | In, Li | P | - | + |  |  |
|  | N-538 | 2010 | 1.5m | Ki | ++ | - | ++ | 1.04X10^7^ |  |
|  |  |  |  | Li, In | P | - | ++ |  |  |
|  |  |  |  | He | - | - | + |  |  |
|  | N-409 | 2016 | 2.5m | Ki | +++ | ++ | ++ | 1.34X10^5^ |  |
|  |  |  |  | He | P | ++ | ++ |  |  |
|  |  |  |  | Sp | P | - | ++ |  |  |
|  |  |  |  | Ad | P | - | + |  |  |
|  |  |  |  | Li | - | - | + |  |  |
|  | N-556 | 2010 | 2m | Ki | +++ | - | +++ | 1.23X10^6^ |  |
|  |  |  |  | Li, He | - | - | + |  |  |
|  | N-440 | 2017 | 1.5m | Me | + | - | + | 5.19X10^4^ |  |
|  |  |  |  | He | P | + | + |  |  |
|  |  |  |  | Sp | - | - | ++ |  |  |
|  |  |  |  | Ly | - | - | + |  |  |
|  |  |  |  | Lu, Ki, Li, Mu, Dig | - | - | - |  |  |
|  |  |  |  | CNS | - | + | - |  |  |
|  | N-515 | 2017 | 1.5m | Ly, Sp | - | - | ++ | 6.70X10^5^ |  |
|  |  |  |  | Lu | - | + | ++ |  |  |
|  |  |  |  | Me, Ki | + | - | + |  |  |
|  |  |  |  | Li, Dig, Ad | - | - | - |  |  |
|  |  |  |  | CNS | - | + | - |  |  |
|  | N-537 | 2017 | 2m | Me | + | - | + | 1.72X10^5^ |  |
|  |  |  |  | Lu | - | + | + |  |  |
|  |  |  |  | Sp, Ly | - | - | ++ |  |  |
|  | N-37 | 2018 | 5d | Sp | P | - | +++ | 4.25X10^7^ |  |
|  |  |  |  | Me | +++ | - | +++ |  |  |
|  |  |  |  | Lu, Ly | - | - | ++ |  |  |
|  |  |  |  | To | - | - | - |  |  |
|  | Cr-215 | 2020 | 2m | In | P | - | + | 5.24X10^2^ |  |
|  | B-121 | 2021 | 3m | Ki | ++ | - | ++ | 3.11X10^5^ |  |
|  |  |  |  | Li | P | - | + |  |  |

| Group | Case ID | Year submitted | Age | Tissue | Periarteritis | Other lesions* | ISH score | qPCR viral load** |  |
| --- | --- | --- | --- | --- | --- | --- | --- | --- | --- |
| Myocarditis | B-2072 | 2001 | 1m | He | - | ++ | - | 1.54X10^2^ |  |
|  | N-515 | 2002 | 4.5m | He | - | + | - | - |  |
|  |  |  |  | Sp, Li, Dig, Ki | - | - | - |  |  |
|  | N-152 | 2004 | 21d | He | - | ++ | - | - |  |
|  |  |  |  | Li, Ki, Sp | - | - | - |  |  |
|  | N-688 | 2006 | 1.5m | He | - | + | - | - |  |
|  |  |  |  | Dig, Ki, Li, Sp | - | - | - |  |  |
|  | N-319 | 2010 | 1.5m | He | - | ++ | - | - |  |
|  |  |  |  | Ki, Li | - | - | - |  |  |
|  | N-385 | 2014 | 21d | He | - | + | - | - |  |
|  |  |  |  | Ki, Dig | - | - | - |  |  |
|  | N-301 | 2016 | 1.5m | He | - | + | - | - |  |
|  |  |  |  | Li, Ki, Ad | - | - | - |  |  |
|  | N-267 | 2017 | 2m | He | - | ++ | ++ | 3.08X10^6^ |  |
|  |  |  |  | Ad, NT | - | - | + |  |  |
|  |  |  |  | Li, Ki | - | - | - |  |  |
|  | N-172 | 2018 | 1m | He | - | + | - | 1.00X10^2^ |  |
|  |  |  |  | Mu | - | - | - |  |  |
|  | B-410 | 2021 | 1m | He | - | ++ | - | - |  |
| Reproductive disease | N-295 | 2009 | A | He, Lu, Sp | - | - | - | - |  |
|  | N-161 | 2002 | A | He, Ki, Sp, Lu | - | - | - | - |  |
|  | B-2968 | 2002 | S | He | P | ++ | +++ | 2.87X10^6^ |  |
|  |  |  |  | Sp | P | - | ++ |  |  |
|  |  |  |  | Li | P | - | + |  |  |
|  | B-723 | 2008 | W | Ly, Sp | - | - | +++ | 8.10X10^7^ |  |
|  |  |  |  | He, Lu | - | - | ++ |  |  |
|  |  |  |  | Ki | - | - | + |  |  |
|  | B-345 | 2010 | A | He | - | + | ++ | 3.70X10^5^ |  |
|  |  |  |  | Sp | - | - | + |  |  |
|  |  |  |  | Li | - | - | - |  |  |
|  | B-1193 | 2010 | A | He, Lu, Ki, Me | - | - | - | - |  |
|  | N-409 | 2011 | S | He, Ki, Lu, Li, Ly | - | - | - | 1.00X10^2^ |  |
|  | N-20 | 2012 | S | He, Lu, Ly | - | - | - | - |  |
|  | N-77 | 2012 | M | He | - | ++ | +++ | 6.43X10^7^ |  |
|  |  |  |  | Ly, Sp | - | - | +++ |  |  |
|  |  |  |  | Ki | - | - | ++ |  |  |
|  |  |  |  | Lu | - | - | + |  |  |
|  | B-924 | 2017 | A | He | - | + | - | - |  |

| Group | Case ID | Year submitted | Age | Tissue | Periarteritis | Other lesions* | ISH score | qPCR viral load** |  |
| --- | --- | --- | --- | --- | --- | --- | --- | --- | --- |
| PDNS | B-587 | 2005 | 3m | Lu, Ki, Ly | - | PDNS | - | - |  |
|  | N-639 | 2005 | 4.5m | Li, Ki, Sp | - | PDNS | - | - |  |
|  | N-644 | 2005 | 4.5m | Sp, He, Ki, Li | - | PDNS | - | - |  |
|  | B-1 | 2006 | 3m | Ki, Li | - | PDNS | - | - |  |
|  | N-10 | 2006 | 3.5m | Ki | - | PDNS | - | - |  |
|  | B-479 | 2007 | 2.5m | Ki, Ly, Lu | - | PDNS | - | - |  |
|  | B-365 | 2012 | 5m | Ki, Sp, To, Lu, Ly | - | PDNS | - | - |  |
|  | B-71 | 2013 | 6m | Ki, Ly | - | PDNS | - | - |  |
|  | B-121 | 2015 | 3m | Ki | - | PDNS | - | - |  |
|  | B-865 | 2018 | 4m | Ki | - | PDNS | - | - |  |
| Encephalitis | N-247 | 1998 | 1.5m | CNS | - | + | - | - |  |
|  | B-807 | 1999 | 4m | CNS | - | + | - | - |  |
|  | B3160 | 2002 | 3m | CNS | - | ++ | - | - |  |
|  | B-524 | 2005 | 3m | CNS | - | + | - | - |  |
|  | B-456 | 2006 | 2m | CNS | - | + | - | - |  |
|  | B-249 | 2011 | 2m | CNS | - | + | - | - |  |
|  | N-127 | 2017 | 1m | CNS | - | + | - | - |  |
|  | N-217 | 2017 | 1m | CNS | - | + | - | - |  |
|  | N-189 | 2018 | 2m | CNS | - | ++ | - | 6.53X10^2^ |  |
|  | B-172 | 2020 | 4m | CNS | - | +++ | - | - |  |
| PFTS | N-169 | 2012 | 28d | CNS | - | + | + | 1.00X10^2^ |  |
|  |  |  |  | Me | + | - | + | 4.99X10^3^ |  |
|  |  |  |  | Ly | - | - | + |  |  |
|  |  |  |  | Th | - | - | - |  |  |
|  | N-133 | 2017 | 45d | Me | + | - | + | 1.00X10^2^ |  |
|  |  |  |  | CNS | - | + | + |  |  |
|  |  |  |  | Lu, Ly, He, Ki, Li, Mu, Ad, NT, dig | - | - | - |  |  |
|  | N-224 | 2016 | 1m | Me | ++ | - | +++ | 1.21X10^7^ |  |
|  |  |  |  | Sp, Ly | - | - | ++ |  |  |
|  |  |  |  | Lu | - | - | + |  |  |
|  |  |  |  | To | - | - | - |  |  |
|  | B-230 | 2014 | 1.5m | Ly | P | - | ++ | 2.60X10^5^ |  |
|  |  |  |  | Dig | - | - | + |  |  |
|  | N-184 | 2019 | 1.5m | Ly, Sp, Dig | - | - | + | 2.26X10^3^ |  |
|  |  |  |  | Lu, Ki, He, NT, Li, Ad, CNS | - | - | - |  |  |
|  | N-200 | 2012 | 1.5m | Lu, To, Ly, He, Ki, Li, Sp, Dig, CNS | - | - | - | - |  |
|  | N-426 | 2017 | 1m | Dig, He, Ki, Li, NT, Dig, Mu, CNS | - | - | - | - |  |
|  | B-877 | 2017 | 2m | He, Ly, Dig, Lu, Ly | - | - | - | - |  |
|  | N-350 | 2018 | 1.5m | Lu, Ly, He, Ki, Li, Dig, Sp. CNS | - | - | - | - |  |
|  | N-191 | 2011 | 1m | Me, Ly, Lu | - | - | - | - |  |

| Group | Case ID | Year submitted | Age | Tissue | Periarteritis | Other lesions* | ISH score | qPCR viral load** |  |
| --- | --- | --- | --- | --- | --- | --- | --- | --- | --- |
| CT | N-660 | 2006 | 1d | CNS | - | + | - | - |  |
|  | N-142 | 2008 | 2d | CNS | - | - | - | 1.00X10^2^ |  |
|  | B-91 | 2010 | 2d | CNS | - | - | - | - |  |
|  | N-496 | 2010 | 1d | CNS | - | - | - | - |  |
|  | N-516 | 2011 | 7d | CNS | - | - | - | - |  |
|  | N-546 | 2011 | 7d | CNS | - | + | - | - |  |
|  | N-266 | 2012 | 2d | CNS | - | + | - | - |  |
|  | N-423 | 2012 | 1d | CNS | - | - | - | - |  |
|  | N-579 | 2011 | 1d | CNS | - | + | - | - |  |
|  | N-293 | 2014 | 7d | CNS | - | + | - | - |  |

*Other lesions considered were non-suppurative myocarditis, interstitial pneumonia, interstitial nephritis and non-suppurative encephalitis in the corresponding organ. Grading of these lesions was – (absence), + (mild), ++ (moderate), +++ (abundant). For congenital tremors group, lesions recorded in CNS consisted of mild vacuolization of cerebellar white matter.

**qPCR viral load results are given in copies for mL of resuspended pellet from deparaffined tissue.

Age: d (days); m (months); A (aborted); S (stillbirth); W (weak born) M (mummified)

Tissue: He: heart; Ki: kidney; In: intestine; Sp: spleen; Li: liver; Ad: adrenal gland; Lu: lung; Ly: Lymph node; To: tonsil; Me: mesenteric arteries; Mu: skeletal muscle; In: intestine; Dig: digestive organs, either stomach, small intestine or large intestine; CNS: central nervous system.

Periarteritis: P (presence), - (absence).
